# Right-Sided Infectious Endocarditis in the Patient With Williams Syndrome: Importance of Recognizing Disease-Specific Pathophysiology in Adults

**DOI:** 10.7759/cureus.85871

**Published:** 2025-06-12

**Authors:** Satoshi Nakano, Hirofumi Saiki, Kanchi Saito, Akira Sato, Yurie Takizawa, Seiko Kuwata, Kotaro Oyama

**Affiliations:** 1 Pediatric Cardiology, Iwate Medical University, Shiwa, JPN; 2 Pediatrics, Michinoku Medical Center on Disability and Health, Shiwa, JPN

**Keywords:** diverticulits, infectious-septic endocarditis, peripheral pulmonary lesions, pulmonary stenosis, williams syndrome

## Abstract

Infectious endocarditis (IE) in patients with Williams syndrome is usually associated with left-sided heart lesions, whereas right-sided endocarditis is rarely observed. This can be explained by the natural course of congenital heart lesions in Williams syndrome, in which right-sided heart lesions are likely to spontaneously regress with patient growth.

We encountered a 29-year-old patient diagnosed with right-sided infective endocarditis. His diagnosis was supported by the modified Duke criteria, one major criterion of positive blood culture twice with *Streptococcus oralis*, a type of gram-positive *viridance Streptococcus*, and three minor criteria of the presence of congenital heart disease, persistent fever higher than 38.0℃, and spatial and temporal dissemination of septic pulmonary emboli. Although his supravalvular aortic stenosis remained mild, peripheral pulmonary stenosis was progressive even after adulthood, which might have been attributed to the development of right-sided infectious endocarditis, based on the biased distribution of septic emboli. Severely advanced caries with extensive tooth decay were not diagnosed until the development of IE because of intellectual disability and inability to report subjective symptoms. The subsequent development of diverticulitis after the treatment of infectious endocarditis was difficult to diagnose, and the management of this patient became more complicated. As the disease-specific pathophysiology progresses with age and adult Williams syndrome patients are less likely to express subjective symptoms, a multidisciplinary approach by the medical team comprised of cardiologists, nephrologists, gastroenterologists, dentists, and psychologists is needed in the management of Williams syndrome, particularly in adults in the process of becoming independent.

## Introduction

Williams syndrome is a microdeletion syndrome of the chromosome 7q11.23, presenting with aortic/pulmonary stenosis and mental retardation during childhood. While infective endocarditis (IE) has been reported in patients with supra-aortic stenosis or mitral valve lesions [1-3], to our knowledge, right-sided heart involvement has not been reported, probably because of the spontaneous remission of pulmonary stenosis [4], and predominant development in the left-sided heart lesions [5]. Here, we report the clinical course of a case of suspected right-sided IE in adult Williams syndrome, which required a multidisciplinary approach by cardiologists, nephrologists, gastroenterologists, dentists, and psychologists because of the disease-specific pathophysiology of this patient.

## Case presentation

A twenty-nine-year-old male patient with Williams syndrome was referred to us due to long-lasting cough. He was initially referred to our hospital because of cyanosis at one week of age and was diagnosed with atrial septal defect (ASD), peripheral pulmonary stenosis (PS), and supravalvular aortic stenosis (AS). He was diagnosed with Williams syndrome during infancy, based on his facial appearance and characteristic cardiovascular phenotype. Since all cardiovascular lesions were mild and his cyanosis disappeared, he was followed up as an outpatient without any therapeutic intervention every two to three years.

**Figure 1:**
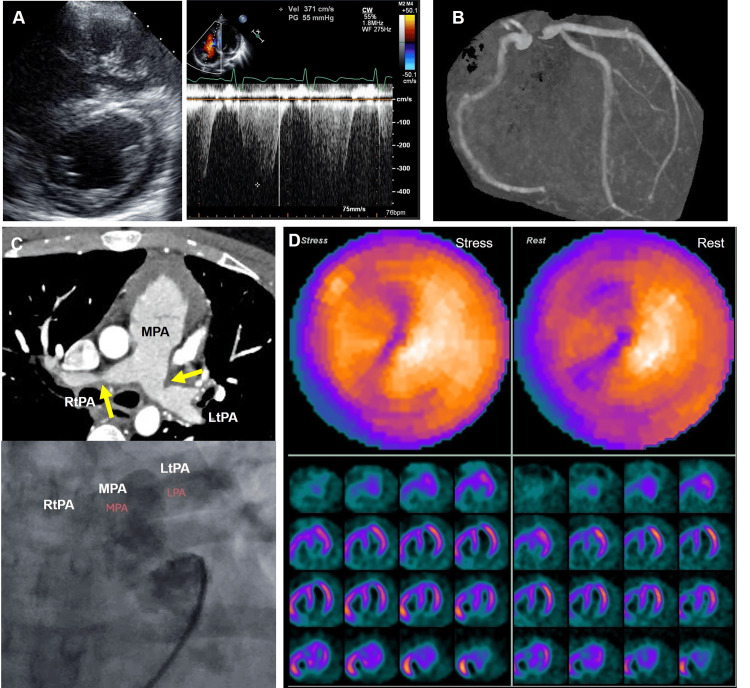
Cardiac Images at 20 years of age A: Echocardiogram at the age of 20 years. Mild pulmonary hypertension was indicated by the shape of the interventricular septum (left panel) and velocity of tricuspid valve regurgitation (right panel). The peak velocity of tricuspid regurgitation was 3.7 m/s, which corresponds to a pressure gradient of 55 mmHg. B: No stenotic lesion is identified in the coronary arteries on computed tomography angiography. C. The computed tomography angiography revealed bilateral pulmonary stenosis (yellow arrows, upper panel). The catheter angiogram revealed antegrade blood flow to the bilateral pulmonary arteries (lower panel). D: Nuclear cardiac perfusion imaging is shown. Neither stressed nor resting images indicated perfusion defects. Abbreviations: LtPA, left pulmonary artery; MPA, main pulmonary artery; RtPA, right pulmonary artery.

At the age of 20 years, an annual cardiac checkup revealed suppressed left ventricular contractility with an ejection fraction of less than 40% and elevated right ventricular pressure (Figure 1A), which could not be evaluated at this point. He reported no clinical symptoms, and computed tomography (CT) angiography revealed no stenotic lesions in the coronary arteries (Figure 1B) or bilateral pulmonary stenosis (Figure 1C, upper panel). His plasma B-type natriuretic peptide level was within the normal range. Cardiac catheterization revealed a Qp/Qs ratio of 1.2, with a right ventricular systolic pressure to left ventricular pressure ratio of 0.6. The systolic pressure gradient between the bilateral pulmonary arteries and the main pulmonary artery was 40 mmHg on the left and 30 mmHg on the right (Figure 1C), indicating that the elevated right ventricular pressure was due to the progression of bilateral pulmonary stenosis. No pressure gradient was observed between the left ventricle (LV) and aortic arch. Contrast imaging also revealed no stenotic lesions in the coronary arteries. Although the left ventricular motion of the inferior and lateral walls appeared to be suppressed in the echocardiogram, stressed perfusion imaging revealed negative results for myocardial ischemia (Figure 1D). Taken together, the patient was on carvedilol and was observed on an outpatient basis without any cardiovascular events for nine years.

**Figure 2:**
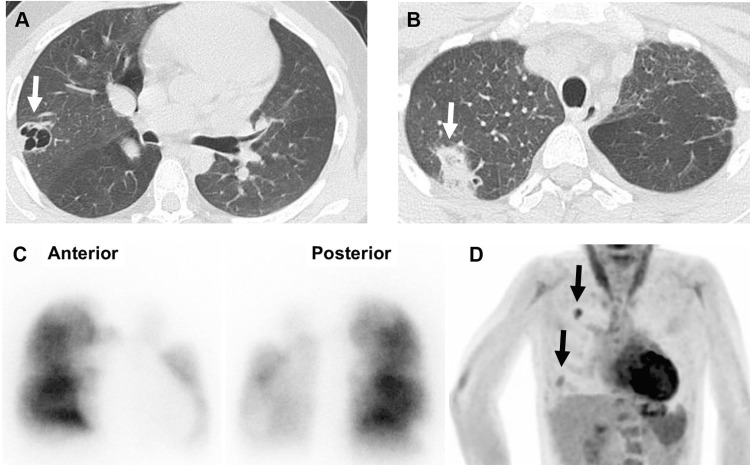
Images at admission A: Computed tomography (CT) indicated vesicles in the right lung (arrow), implying the possibility of pneumonia, although we needed to differentiate this from the other pulmonary diseases. This patient was negative for tuberculosis or fungal infection. B: Follow-up CT image after three months is shown. Although the original vesicle observed in panel A disappeared, other consolidation lesions with vacuoles were identified (arrow). C: Pulmonary blood flow scintigraphy showing marked disappearance of left lung perfusion. D: ^18^F-fluorodeoxyglucose-positron emission tomography revealed no signals in the heart or other organs, and only multiple hot lesions in the right lung were observed (arrows).

Four months before his referral to us, he started to exhibit cough and fever temporarily, and CT imaging identified possible pneumonia (Figure 2A), requiring differentiation from other lung diseases. Since he was negative for tuberculosis, he was empirically treated with tosufloxacin (TFLX). His condition markedly improved and he was able to continue his part-time jobs without admission. Although his condition improved with antibiotics, symptoms including fever, persistent cough, and weight loss recurred with its discontinuation, and he was admitted to our hospital for further examination. He exhibited a mild fever; otherwise, his general condition was excellent. The lung CT image showed two separate lesions of consolidation with vacuoles (Figure 2B), which implied the possibility of septic emboli. Both transthoracic and transesophageal echocardiograms were negative for cardiac vegetation, and no worsening of the valvular regurgitation was observed. The LV ejection fraction was 47% with an estimated right ventricular pressure of 67 mmHg, which was similar to that observed at 20 years of age. Cardiac catheterization revealed a Qp/Qs of 1.25 due to left-to-right shunting of the ASD, with a right ventricular pressure of 48 mmHg and mean pulmonary pressure of 24 mmHg. Although the pressure gradient between the main and right peripheral pulmonary arteries was 41 mmHg, the pressure gradient between the main and left peripheral pulmonary arteries was not assessed because of the wedge pressure waveform, implying exacerbation of the left peripheral PS. While contrast imaging was unavailable because of compromised renal function originating from the ectopic kidney with an estimated glomerular filtration rate of 27 ml/m^2^/min, this was further supported by the findings of pulmonary blood flow scintigraphy showing an almost complete left lung defect (Figure 2C), where antegrade contrast was identified during cardiac catheterization at 20 years of age (Figure 1C, lower panel), demonstrating progression of the left peripheral PS to a semi-complete obstruction. Although *Streptococcus oralis* was repeatedly identified in the blood culture at admission, no signals in the heart or other organs were identified on ^18^F-fluorodeoxyglucose-positron emission tomography (Figure 2D), except for multiple lesions in the right lung, which were different locations from the signals observed in the previous CT imaging. Taken together, he was, by definition, diagnosed with definitive IE by one major criterion of positive blood culture twice with *Streptococcus viridance* (a Gram-positive *viridans Streptococcus* of the *Streptococcus mitis group*), and three minor criteria for the presence of congenital heart disease (peripheral pulmonary stenosis), persistent fever higher than 38.0℃, and spatial and temporal dissemination of septic pulmonary emboli according to the modified Duke criteria. Thus, we suspected peripheral right pulmonary artery stenosis as the origin of infection based on the temporal and spatial dissemination of septic emboli limited to the right lung and negative findings inside the heart. Unfortunately, the time series of peripheral PS progression, development of IE, and formation of septic emboli could not be confirmed because of the empiric administration of oral antibiotics before referral to our hospital, which might have hindered us from obtaining definitive evidence of pathological IE.

**Figure 3:**
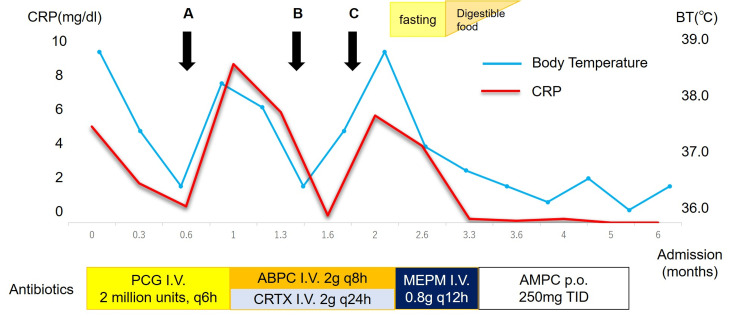
Clinical course after admission The patient’s clinical course after admission is presented. A: Multiple blood cultures were negative for bacteria. B: Consultation with dentists and treatments for caries. C: Diverticulitis was suspected and endoscopy was performed. Abbreviations: ABPC, ampicillin; AMPC, Amoxicillin; CTRX, Ceftriaxone; I.V., intravenous administration; MEPM, meropenem hydrate; PCG, penicillin G; p.o., oral administration.

**Figure 4:**
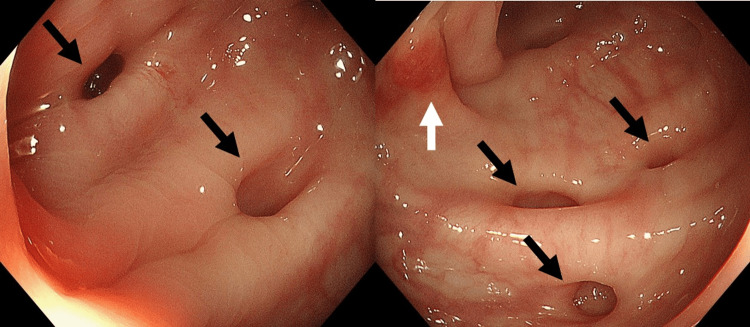
Gastrointestinal endoscopy Gastrointestinal endoscopy revealed multiple lesions of diverticulosis (black arrow) and mild inflammation (white arrow).

As *S. oralis* identified in the blood culture was susceptible for most of the antibiotics except for clarithromycin and azithromycin, we started penicillin G infusion at a dose of 2 million units every six hours, based on the compromised renal function dosage. He became afebrile, and the serum inflammatory marker of C-reactive protein (CRP) was negative (Figure 3, arrow A). However, his fever again resulted in a marked increase in CRP level in three weeks of antibiotics; thus, the antibiotics were upgraded to ampicillin (2 g, every eight hours) and ceftriaxone (2 g, every 24 hours). As the blood culture was negative at this point, we consulted dentists, who revealed multiple, severely advanced caries with extensive tooth decay (Figure 3, arrow B). The patient was treated concurrently, and the fever resolved. Despite these efforts, the patient became febrile with elevated serum CRP levels within five weeks. Although his cough disappeared at this point and CT imaging demonstrated no apparent pulmonary infiltration, his brow darkened repeatedly during abdominal palpation and he vomited a few times. Based on the diagnosis of Williams syndrome, we suspected that this was a manifestation of diverticulitis. As expected, gastrointestinal endoscopy identified multiple diverticulosis with suspected mild inflammation in the colon (Figure 3, arrow C; Figure 4). As the six-week treatment regimen for IE was almost complete, and as a diagnosis of diverticulitis was made, we regarded that IE was likely to be treated successfully. Accordingly, we converted the antibiotics to meropenem hydrate at a dose of 800 mg every 12 hours, which covers both *S. oralis* and gram-negative bacterial species, and abstained from eating, with a tentative diagnosis of diverticulitis. He became afebrile again, and inflammatory marker levels were within the normal range. After three weeks of meropenem hydrate infusion, it was switched to oral amoxicillin, and the patient was discharged from the hospital. Oral amoxicillin was prescribed as a post-therapy and discontinued within two months. He started a routine checkup with dentists, nephrologists, and gastroenterologists every three months, in addition to the clinical visit to a cardiologist.

## Discussion

Reported cases of IE in patients with Williams syndrome have been only left-sided cardiac involvement associated with supravalvular aortic stenosis and mitral regurgitation [1-3]. Meanwhile, there is a theoretical risk of developing right-sided IE when pulmonary artery stenosis progresses with the development of right ventricle (RV) pressure elevation, even though the risk of developing right-sided IE is low in the general population [5]. In our case, the bilateral peripheral PS was initially unremarkable, but it progressed at the age of 20 years with the elevation of right ventricular pressure, which further exacerbated the left pulmonary stenosis to almost atretic unilaterally at the age of 29 years. Although there was no apparent vegetation, we speculated that preferential blood flow to the right lung might have increased the shear stress to the right peripheral arteries, which probably contributed to the development of clinical infectious endocarditis. Disease-specific physiology, including mental retardation, difficulty in expressing subjective symptoms, and fragility of systemic elastic fibers in the systemic organs, further complicated therapeutic interventions in this case.

Although peripheral pulmonary stenosis in patients with Williams syndrome is reported to regress with patient growth [4], our patient worsened after 20 years of age and almost occluded unilaterally at the age of 29, highlighting the importance of following up these cohorts even after adulthood. The reason for progression in peripheral PS is unclear, but the reported cases of spontaneous remission have been in a pediatric cohort, and further investigation in adults is warranted [4]. The reported cases of infectious endocarditis in Williams syndrome are only left heart lesions [1-3]. Progressive peripheral pulmonary stenosis and a subsequent increase in right ventricular pressure might have been related to the development of IE, although stenosis in the right heart system is generally considered to be low risk [6]. Williams syndrome is not usually associated with immunodeficiency and is not particularly susceptible to IE. However, the patient's mild temperament and developmental delay made it difficult for him to report symptoms; thus, the presence of dental caries was not noticed until he developed IE. The diagnosis of infective endocarditis was made based on modified Duke's criteria [7], and indeed, we could not identify the actual vegetation in the right heart system, although septic emboli were identified only in the right lung. Despite interatrial communication with elevated right ventricular pressure, no systemic septic emboli or abscesses were found aside from the lungs, supporting the idea that the focus of IE is the right peripheral pulmonary artery. Indeed, septic pulmonary emboli are a manifestation that prompts the search for right-sided IE [8]. Due to the lack of visualized vegetation, the diagnosis of IE is not pathological, but based on the clinical definition of the modified Duke criteria. Although we could not determine when he developed bacteremia, his tooth caries were multiple, severely advanced with extensive tooth decay, supporting tooth as the source of bacteremia. Renal dysfunction hindered the additional use of contrast in imaging, which might have limited the ruling out the possibility of an embolic source other than the peripheral pulmonary artery.

Another reason for the difficulty in managing this case was the development of diverticulitis during treatment. Williams syndrome is a genetic abnormality associated with elastin gene, and diverticulosis is reported to be identified as high as 18% in the pediatric cohort [9]. As the prevalence of diverticulosis markedly increases with age, it is not surprising that multiple diverticulosis develops as early as at the age of 29 years in Williams syndrome with elastic fiber abnormality. The onset of diverticulitis is closely related to the use of antibiotics in the past four years, with significant association to the length of its use [10]. Prolonged use of antibiotics for more than three months coupled with multiple diverticulosis might be associated with the development of diverticulitis. Patients with Williams syndrome are less likely to attract attention or express anxiety either due to mental retardation and mild temperament, which made them face difficulty in living as independent adults [11]. Since it has been reported that patients with Williams syndrome lack subjective symptoms, specialized care should be taken in the diagnosis and treatment of this cohort [11]. While our case was successfully treated with conservative treatments, the reported cases of diverticulitis required urgent surgical intervention due to perforation [12], which might be associated with the inability to report symptoms in patients with Williams syndrome.

## Conclusions

We reported a case of right-sided infective endocarditis in an adult with Williams syndrome. Although the left-heart cardiac lesion was mild, the right-heart lesion after adulthood was progressive, which is speculated to be the cause of IE. Due to intellectual disability and inability to report subjective symptoms in this cohort, the development of diverticulitis during the treatment of IE was difficult to diagnose, and its management became more complicated. Cardiologists who participate in the management of adult congenital heart disease should be fully aware of and advised to investigate symptoms related to systemic complications, including dental caries and diverticulitis, beyond cardiac lesions in patients with Williams syndrome. A multidisciplinary approach should be implemented by an organized medical team comprising cardiologists, nephrologists, gastroenterologists, dentists, and psychologists to address the pathophysiology of Williams syndrome, particularly in adults in the process of becoming independent.
